# Postoperative acute pancreatitis after pancreatoduodenectomy: the importance of considering modifiers when validating a definition

**DOI:** 10.1093/bjsopen/zrac045

**Published:** 2022-04-26

**Authors:** Saxon Connor

**Affiliations:** Department of Surgery, Canterbury District Health Board, Christchurch, New Zealand

Postoperative acute pancreatitis (POAP) has become a point of interest among pancreatic surgeons with the increasing recognition of its aetiological role in major postoperative morbidity following pancreatoduodenectomy (PD). Bonsdorff *et al.* present a retrospective series of 614 patients who underwent pancreatoduodenectomy between 2013 and 2020 at Helsinki University Hospital.^1^ The aim of this study was two-fold, first to reconfirm the impact of clinically relevant POAP (CR-POAP) on early outcomes following PD but also to assess the utility of presently used fistula risk scores in predicting CR-POAP. It is acknowledged that the definition of CR-POAP remains an area of evolution^2,3^ and for this study the authors elected to use the initial definition proposed by my previous work (serum amylase above upper limit of normal day 0–1 and C-reactive protein (CRP) more than180 mg/dl day 1–2)^2^. Importantly it should be noted that somatostatin analogues and hydrocortisone were selectively used for patients defined as having a high-risk remnant, with some being enrolled in a randomized clinical trial comparing the aforementioned agents. No information was provided regarding external pancreatic stent use. Complications were reported by use of the comprehensive complication index (CCI). Patients were split into those with high (CCI score 33.7 or higher) and low (CCI less than 12.3) morbidity. Of the 614 patients who met inclusion criteria, 106 (17 per cent) were excluded due to missing data regarding CR-POAP. No information is provided regarding the outcomes of those patients. Neoadjuvant therapy was administered to 106 (21 per cent) of included patients. Data on patients receiving hydrocortisone or somatostatin analogues are not provided and do not seem to have been considered as potential variables in the outcome analyses. In addition, it is difficult to determine the distribution of patients across the fistula risk-score zones, although data on the individual components are provided. In terms of outcomes, patients with CR-POAP made up 39 per cent of patients with high morbidity, but only 4 per cent of those patients with low morbidity, thus confirming the importance of this complication. Of the 64 patients diagnosed with CR-POPF, 46 (72 per cent) had underlying CR-POAP, but only 4 patients with CR-POPF had CRP more than 180 ng/dl on day 2 and a normal day 1 amylase. The updated alternative fistula risk score^4^ seemed to best predict both CR-POPF and CR-POAP with an area under the curve (95 per cent c.i.) of 0.819 (0.771 to 0.867) and 0.834 (0.794 to 0.873) respectively.

This paper adds further weight to the increasing body of evidence that POAP is a major aetiological factor in pancreatic specific morbidity following pancreatoduodenectomy. However, this paper also highlights that while the definition of POAP continues to be refined or validated, it will be important to consider how modifying interventions may alter the outcomes and thus alter the accuracy of the assessment of the definition in question. There is now evidence that hydrocortisone attenuates postoperative inflammatory response and morbidity.^5^ In addition, external stenting, and omission of somatostatin analogues are considered to be the optimal mitigation strategy for high-risk anastomoses^6^. Thus, it is possible such interventions will ‘down stage’ the severity of the complication, thus biasing assessment, or validation of the definition. The increasing use of neoadjuvant therapy is also another contributing factor, as this almost certainly modifies the underlying pancreatic remnant. Such patients rarely develop CR-POAP, yet the overall complication burden may well be higher due to the more extensive surgery; in this series alone, 3 per cent of patients underwent arterial resection. Thus, it may become important when validating and refining the burden of POAP that the analysis is stratified by the state of the underlying pancreatic remnant. It is highly likely that the underlying inflammatory response and risk of pancreatic necrosis is highly variable between such patients.

*Disclosure*. The authors declare no conflict of interest.

## References

[1] Bornsdorff A , Helantera I, Tarvainen T, Siren J, Kokkola A, Sallinen V. Prediction and consequence of post-operative pancreatitis after pancreatoduodenectomy. BJS Open 2022;6:zrac012 10.1093/bjsopen/zrac012 PMC9039121 35470380

[2] Connor S . Defining post-operative pancreatitis as a new pancreatic specific complication following pancreatic resection. HPB 2016;18:642–651 27485058 10.1016/j.hpb.2016.05.006 PMC4972444

[3] Marchegiani G , Barreto SG, Bannone E, Sarr M, Vollmer CM, Connor S et al Post-pancreatectomy acute pancreatitis (PPAP): definition and grading from the International Study Group for Pancreatic Surgery (ISGPS). Ann Surg 2022;275:663–672 34596077 10.1097/SLA.0000000000005226

[4] Mungroop TH , Klompmaker S, Wellner UF, Steyerberg EW, Coratti A, D’Hondt M et al Updated alternative fistula risk score (ua-FRS) to include minimally invasive pancreatoduodenectomy: pan-European validation. Ann Surg 2021;273:334–340 30829699 10.1097/SLA.0000000000003234

[5] Kuan LL , Dennison AR, Garcea G. Outcomes of peri-operative glucocorticosteroid use in major pancreatic resections: a systematic review. HPB 2021;23:1789–1798 34593313 10.1016/j.hpb.2021.07.001

[6] Ecker BL , McMillan MT, Asbun HJ, Ball CG, Bassi C, Beane JD et al Characterization and optimal management of high-risk pancreatic anastomoses during pancreatoduodenectomy. Ann Surg 2018;267:608–616 28594741 10.1097/SLA.0000000000002327

